# HPV genotyping by L1 amplicon sequencing of archived invasive cervical cancer samples: a pilot study

**DOI:** 10.1186/s13027-022-00456-w

**Published:** 2022-08-09

**Authors:** Charles D. Warden, Preetam Cholli, Hanjun Qin, Chao Guo, Yafan Wang, Chetan Kancharla, Angelique M. Russell, Sylvana Salvatierra, Lorraine Z. Mutsvunguma, Kerin K. Higa, Xiwei Wu, Sharon Wilczynski, Raju Pillai, Javier Gordon Ogembo

**Affiliations:** 1 Integrative Genomics Core, City of Hope National Medical Center, Duarte, CA 91010 USA; 2 Department of Medicine, Vanderbilt University Medical Center, Nashville, TN 37212 USA; 3 Molecular Pathology Core, City of Hope National Medical Center, Duarte, CA 91010 USA; 4 Research Informatics, City of Hope National Medical Center, Duarte, CA 91010 USA; 5 Clinical Informatics, City of Hope National Medical Center, Duarte, CA 91010 USA; 6 Biorepository, City of Hope National Medical Center, Duarte, CA 91010 USA; 7 Department of Immuno-Oncology, City of Hope National Medical Center, Duarte, CA 91010 USA; 8 Office of Faculty and Institutional Support, City of Hope National Medical Center, Duarte, CA 91010 USA; 9 Department of Pathology, City of Hope National Medical Center, Duarte, CA 91010 USA

**Keywords:** Human papillomavirus, Genotyping, High-throughput sequencing, Invasive cervical cancer

## Abstract

**Background:**

Human papillomavirus (HPV) is the primary cause of invasive cervical cancer (ICC). The prevalence of various HPV genotypes, ranging from oncogenically low- to high-risk, may be influenced by geographic and demographic factors, which could have critical implications for the screening and prevention of HPV infection and ICC incidence. However, many technical factors may influence the identification of high-risk genotypes associated with ICC in different populations.

**Methods:**

We used high-throughput sequencing of a single amplicon within the HPV L1 gene to assess the influence of patient age, race/ethnicity, histological subtype, sample type, collection date, experimental factors, and computational parameters on the prevalence of HPV genotypes detected in archived DNA (n = 34), frozen tissue (n = 44), and formalin-fixed paraffin-embedded (FFPE) tissue (n = 57) samples collected in the Los Angeles metropolitan area.

**Results:**

We found that the percentage of off-target human reads and the concentration of DNA amplified from each sample varied by HPV genotype and by archive type. After accounting for the percentage of human reads and excluding samples with especially low levels of amplified DNA, the HPV prevalence was 95% across all ICC samples: HPV16 was the most common genotype (in 56% of all ICC samples), followed by HPV18 (in 21%). Depending upon the genotyping parameters, the prevalence of HPV58 varied up to twofold in our cohort. In archived DNA and frozen tissue samples, we detected previously established differences in HPV16 and HPV18 frequencies based on histological subtype, but we could not reproduce those findings using our FFPE samples.

**Conclusions:**

In this pilot study, we demonstrate that sample collection, preparation, and analysis methods can influence the detection of certain HPV genotypes and must be carefully considered when drawing any biological conclusions based on HPV genotyping data from ICC samples.

**Supplementary Information:**

The online version contains supplementary material available at 10.1186/s13027-022-00456-w.

## Background

Human papillomavirus (HPV) is the primary cause of invasive cervical cancer (ICC) [1]. Although the Papanicolaou (Pap) cytological screening test, improved treatment of ICC precursor lesions, and the development of HPV vaccines [2, 3] have led to a general decline in the incidence and mortality of ICC over the past three decades, additional methods to improve HPV detection are needed to fully eliminate ICC diagnoses and deaths [4–6]. In particular, there is a need to identify HPV genotypes associated with pre-malignant cervical intraepithelial neoplasia of grade 1–3 (CIN1–3) that is likely to progress to ICC [7]. In most cases, HPV infections in women who are HPV−positive but with normal cytology (HPV + /CIN-) are transient and cleared by the host immune system within 12 months [8–10]. However, this is not true for all cases, and the underlying risk factors responsible for the development of CIN3 and subsequently ICC in these women remain largely unknown. Moreover, although the HPV genotypes responsible for most ICC cases are known, certain populations may be particularly susceptible to novel oncogenic HPV genotypes that are rare in more commonly studied populations. To begin to address these gaps in knowledge, we aimed to identify HPV genotypes in archived ICC tumor biopsies, ultimately to improve their detection in HPV + /CIN- women and further decrease ICC incidence.

HPV is a double-stranded circular DNA virus containing approximately 8,000 base pairs (bp) and harboring at least eight open reading frames that encode six functional early proteins (E1, E2, E4, E5, E6, and E7) and two late capsid proteins (L1 and L2) [11–13]. Conventionally, a unique HPV genotype is recognized if the sequence of its L1 gene differs from that of the closest known genotype by > 10%. Of the HPV genotypes currently known to infect humans, several have been reported as high-risk, probably carcinogenic, or possibly carcinogenic in multiple studies and by the International Agency for Research on Cancer (IARC), such as HPV16, 18, 26, 31, 33, 35, 39, 45, 51, 52, 53, 56, 58, 59, 66, 68, 73, and 82 [14–17].

HPV16 is the most common HPV genotype in women with normal cytology [18], as well as in women with ICC, followed by HPV18. Together, HPV16 and HPV18 have been identified in 65–77% of ICC cases worldwide [19]; however, the next most common HPV genotypes and their relative frequencies, as well as their outcomes, vary by continent. Variability across smaller geographical regions has also been demonstrated and may even be greater than continent-based differences. For example, previous studies have shown that HPV58 is associated with a higher risk of developing CIN3 and ICC in East Asia and Latin America than in other regions [20]. Furthermore, HPV genotype diversity also varies depending on cytology. In a multicenter study in Korea, HPV58 was found in 10.8% of all abnormal cytological specimens, making it second only to HPV16 as the most common genotype in women with abnormal cytology [21]; this prevalence, however, was not observed in women with normal cytology. The frequency of HPV16 and HPV18 have also been differentially associated with different histological subtypes of ICC [19, 22, 23].

These observations indicate that commonly used genotype-specific testing for HPV16 alone or concurrently with HPV18 is an incomplete screening strategy for HPV + /CIN- women, particularly in regions with greater racial and ethnic diversity, which may contribute to the persistent disparities in HPV outcomes related to ethnic diversity, socioeconomic status, and geographical location [24–26]. For example, in Los Angeles County where ICC incidence per 100,000 women varies by ethnicity and, for some populations, may be higher than the U.S. national average of 7.7 per 100,000 women: 6.5 among Asian/Pacific Islander women, 7.3 among white women, 8.0 among Latinas, and 10.5 among black women [27]. It was also previously reported that the prevalence of some HPV genotypes varies with patient age [16, 28]. Furthermore, socioeconomic factors have an important influence on health disparities for cervical cancer [29]. A meta-analysis of subjects in the United States specifically looked at HPV genotype variation between race and ethnicity [30], which emphasizes the value of similar studies while also providing context with respect to the expected magnitude of HPV genotype variation.

Hence, in this pilot study, we initially sought to determine how the prevalence of high-risk HPV genotypes among patients treated for ICC in the Los Angeles metropolitan area varies based on patient age, race/ethnicity, and histological subtype. However, as the study progressed, we began to recognize the limitations of our dataset, including small sample sizes and inadequate patient data availability, as well as the complexities of HPV genotyping methods. Thus, to improve the design of future studies to rigorously assess the effects of patient characteristics on the prevalence of HPV genotypes in ICC samples, we shifted our focus to also evaluate the use of low-density genotyping array data to estimate the genetic ancestry of patients and explore the potential effects of archived ICC sample preparation, collection date, and analysis methods on our HPV genotyping results.

Various methods exist for HPV detection and genotype identification [31]. In addition to using genotype-specific primers/probes, low-throughput methods to assign HPV genotypes include PCR amplification using conserved consensus primers, followed by band detection via gel electrophoresis and/or oligonucleotide hybridization [32, 33] or restriction fragment length polymorphism (RFLP) analysis [34]. Lower throughput methods are also capable of detecting co-infections of multiple high risk HPV types [35, 36]. There are several options for HPV detection using whole-genome sequencing, such as tiling small amplicons either for HPV16 specifically [37, 38] or for multiple HPV types [39], using a limited number of large amplicons [40], and conducting whole-genome sequencing without viral enrichment [41, 42]. However, these strategies are associated with higher costs, and only variable portions of the genome are informative for HPV genotyping. Therefore, in this study, we assigned HPV genotypes to archived ICC samples using low-to-intermediate cost, high-throughput sequencing of a single amplicon within the HPV L1 gene, a method similar to those previously evaluated by Yi et al. [43] and Bik et al. [44]. We analyzed three different types of archived samples: DNA previously extracted from fresh frozen tissue, frozen tissue, and formalin-fixed paraffin-embedded (FFPE) tissue. These included a subset of tumor samples collected from the same patient and preserved using more than one archive method.

## Materials and methods

### Sample pathology, archiving, and DNA extraction

Samples were obtained from patients treated for ICC in the Los Angeles metropolitan area and archived at City of Hope. To classify samples as ICC (versus pre-malignant CIN, for example), at least ten consecutive 5-µM tissue sections were cut for histological analysis. The first and last sections from each sample were stained with hematoxylin and eosin (H&E) and were graded according to routine histological analysis by a City of Hope pathologist. For the archived DNA samples, which were initially processed by the City of Hope Pathology Department, methods for DNA extraction from fresh frozen tissue, as well as previous PCR and Southern Blot HPV genotyping results, are described in a prior publication [32]. DNA extractions from archived frozen and FFPE tissue samples were performed by the City of Hope Pathology Core. The Qiagen QIAamp DNA Mini Kit was used for frozen tissues, and the Qiagen GeneRead DNA FFPE Kit was used for FFPE tissues. Archived DNA samples were stored at − 80 °C, frozen tissue samples were stored at − 20 °C, and FFPE sections were stored at room temperature.

Replicate tumor samples from the same patient were processed to test the reproducibility of HPV L1 genotype read fractions. There were three different types of tumor replicates among our specimens: 1) replicate FFPE samples (n = 2); 2) specimens preserved as both frozen and FFPE samples (n = 7); and 3) archived DNA samples paired with frozen samples (n = 3). DNA samples could not be paired using patient records or other metadata, so DNA-frozen tissue pairs were matched based on QC Array Identity-By-Descent (IBD) estimations. QC Array data were not available for FFPE samples, so any pairs that included FFPE samples could only be matched using patient records. One frozen sample from a frozen-FFPE pair was matched to an archived DNA sample using QC Array data, creating a trio of matched samples. Frozen tumor-normal pairs from the same patient were also available (n = 4), identified using patient records and validated using QC Array data. Tumor samples from two tumor-normal pairs had matching FFPE samples, creating two trios of matched tumor/normal samples.

### Illumina HiSeq2500 sample preparation

Amplification of the L1 sequence and library preparation for all samples were performed by the Integrative Genomics Core of the Beckman Research Institute of City of Hope. L1 amplicons were amplified using GP5 + forward (TTTGTTACTGTGGTAGATACTAC) and GP6 + reverse (GAAAAATAAACTGTAAATCATATTC) consensus primers that detect an approximately 150-bp region of the HPV L1 gene [45]. 50-μl PCR reactions were performed, each containing 50 ng of purified DNA (calculated using a Qubit fluorometer), 0.2 mM of dNTPs, 1.5 mM of MgCl_2_, 1.25 U of Platinum Taq DNA polymerase, 2.5 μl of 10X PCR buffer, and 0.5 μM of each primer. Cycling conditions were: denaturation at 95 °C for 5 min; touchdown annealing at 95 °C for 1 min, followed by 55 °C to 40 °C for 2 min in 1.0 °C decrements (16 cycles); additional annealing cycles at 40 °C for 2 min (10 cycles); and elongation at 72 °C for 5 min. Amplicons were purified using 6% polyacrylamide gel electrophoresis (PAGE), followed by gel extraction. PCR products were quantified using a Qubit fluorometer, and up to 15 ng was used for library preparation in a second round of PCR. The Illumina primer PCR PE1.0 and index primers were used to allow multiplexing of samples. Eight cycles of enrichment PCR were performed, and final libraries were cleaned using an AxyPrep Mag PCR Clean-up kit.

DNA concentrations (used for the “qPCR filter”) were quantified by real-time quantitative PCR (qPCR) using the Applied Biosystems (ABI) ViiA™ 7 Real-Time PCR System (Life Technologies) and visualized for size validation on an Agilent 2100 Bioanalyzer (Agilent Technologies). The 10-µL qPCR reaction system contained 2 µL of 50X-diluted library DNA or a standard library control, 10 pmol forward (5ʹ AATGATACGGCGACCACCGAGAT 3ʹ) and reverse (5ʹ CAAGCAGAAGACGGCATACGA 3ʹ) primers, nuclease-free water, and 5 µL of 2X KAPA SYBR® FAST qPCR Master Mix (Kapa Biosystems). The qPCR program consisted of pre-incubation at 95° for 3 min, followed by 20 cycles of denaturation at 95 °C for 3 s and annealing at 60 °C for 30 s. The quantification cycle (Cq) value represents the number of cycles needed to reach a set threshold SYBR Green fluorescence signal level, which is a measure of the number of cDNA or DNA copies. The calculation of the initial concentration of library templates was based on a standard curve generated from control template dilutions.

### Illumina HiSeq2500 de-multiplexing and FASTQ generation

Image analysis was performed using Real-Time Analysis software v2.2.38, and base calling was performed using bcl2fastq v1.x (could be verified as v1.8.4 for FFPE L1 amplicon samples processed in 2017, but.bcl files used to de-multiplex archived DNA and frozen L1 amplicon samples in 2016 and earlier were unrecoverable). Runs 244, 256, and 271–273 were performed using a machine that was purchased as a HiSeq2500 (D00579). Run 416 was performed using a machine that was purchased as a HiSeq2000 and upgraded to a HiSeq2500 (SN667).

Cross-contamination between HPV L1 amplicon samples is hard to estimate, but exact matches to HPV L1 amplicon sequences can provide a measure of cross-contamination *into* other samples. When available, those results and additional de-multiplexing information can be accessed within “*Cross-Contamination Into Samples From Other Labs*” in [46].

### HPV L1 amplicon read processing

Primer sequences were removed using cutadapt [47], and reads were aligned to a joint reference set, including the human genome (hg38) and a 35-HPV genotype reference set (described below), using BWA-MEM [48]. This joint reference alignment is conceptually similar to what was described by Conway et al. [41].

### HPV genotype reference set development

We developed our HPV genotype reference set through an iterative process. After defining a preliminary reference set including the HPV genotypes provided by Muñoz et al. [16], we tested its suitability for each batch of samples (one batch each of archived DNA and frozen tissue samples, and three batches of FFPE samples analyzed at the same time). Samples with low overall HPV alignment rates using our preliminary reference set were further assessed by merging paired-end reads using PEAR (Paired-End reAd mergeR) [49], identifying overrepresented sequences using FastQC [50], and using BLAST [51] to query the full nucleotide database for sequences present with read fractions > 5%. We also incorporated reference sequences found in additional reviews that we identified as we added and queried unique read sequences throughout the process. This process was repeated until the number of HPV sequences in our reference set ceased to grow. Our final reference set of 35 HPV genotypes included those commonly reported as high-risk for ICC, as well as others reported as low-risk or whose risk level remains unclear: HPV6(b), 11, 16, 18, 26, 30, 31, 33, 34, 35, 39, 40, 42, 43, 44, 45, 51, 52, 53, 54, 56, 58, 59, 61, 66, 67, 68, 69, 70, 72(b), 73, 81, 82, 85, and 97 [14, 16, 17] (Additional File 1: Table S1).

We also used the PaVE database [59] to increase the number of HPV genotypes in the joint reference set to 220 and observed no differences in the genotyping assignments made compared to our 35-genotype reference set (“*PaVE Reference Comparison*” in [46]).

### Illumina QC array sample preparation

Illumina Infinium QC Array analysis was conducted by the Integrative Genomics Core using the Illumina Infinium QC Array-24 Kit, according to Illumina’s Infinium HTS Assay Reference Guide (15,045,738-A). First, all archived DNA and frozen tissue samples were processed using the Infinium HD FFPE Restore Kit. DNA was then amplified, fragmented, precipitated, resuspended in RA1 buffer, and hybridized to Infinium QC Array-24 BeadChips overnight at 48 °C. After hybridization, each BeadChip was washed, stained, signal-extended, and coated, then scanned using the Illumina HiScan.

### QC array processing and genetic similarity calculations

QC Array genotypes were called using Illumina GenomeStudio v2.0.3 with cluster file ‘Infinium QC Array-24v1-0_A3_ClusterFile.egt’ and annotation file ‘Infinium QC Array-24v1-0_A3.bpm.’ We assessed QC Array data using plink to identify IBD segments and validate sample identities [52]. For comparison, we used twenty 1000 Genomes Project trios (family IDs: 2436, CLM27, CLM42, IBS015, IBS060, m002, PEL014, PR08, PR31, SH001, SH014, SH025, SH064, Y019, Y028, Y044, Y056, Y105, Y116, and Y120), which were genotyped with the Omni 2.5 M chip that shares 10,442 matching probes with the QC Array [53]. Prior to the IBD calculation, we pruned matching probes in linkage disequilibrium using the ‘indep-pairwise’ function of plink, with a window size of 50 kb, step size of 5, and r2 of 0.2. Parent-sibling pairs were expected to have proportion IBD (PI_HAT) values greater than 0.4. QC Array samples from the same individual could be identified with PI_HAT values greater than 0.95, which is comparable to the “self” concordance metric reported in another study using the QC Array [54].

### QC array super-population assignments

Data from the 1000 Genomes Project was used as a gold standard for assigning samples to African (AFR), East Asian (EAS), European (EUR), admixed American (AMR), and South Asian (SAS) super-populations [53]. We first assigned samples to super-populations using ADMIXTURE [55] and then calculated confidence values for assignments to each reference set, based on distance to median allele counts, using a separate bootstrap simulation (1,000 bootstraps per sample). Code for QC Array super-population assignments can be accessed in [56].

### Statistical analysis of HPV genotype frequency variation

Paired-end read counts were calculated using samtools [57] idxstats. HPV− samples and HPV genotypes not represented in at least 1% of samples were excluded prior to statistical analysis. Unless otherwise noted, samples with amplified DNA concentrations less than 2 nM (quantified by qPCR after gel extraction) were also excluded from our analysis. Criteria used for HPV genotype assignments were: a minimum HPV genotype read fraction of 20%, overall HPV reads > 1.2 × human reads, and genotype-specific HPV reads > 1.0 × human reads.

To analyze the effects of archive type (FFPE versus DNA, FFPE versus frozen, and frozen versus DNA), samples were compared using Fisher’s exact test. Tests for the effects of age and collection date were similar, except 50 years of age and the year 2000 were used as the thresholds for 2-group analysis. Age and collection year were not available for the archived DNA samples; thus, these analyses only considered frozen and FFPE samples. To analyze the effects of super-population, only EUR and AMR data were compared. To analyze the effects of histological subtype, squamous cell carcinoma (SCC) samples were compared to the combined group of adenocarcinoma (AC) and adenosquamous carcinoma (ASC) samples (“Adeno types”) using HPV genotype assignments made based on a 20% minimum read fraction and excluding samples with low amplified DNA concentrations (< 2 nM; i.e., including only samples “passing the qPCR filter”), as well as assignments made using a 5% minimum read fraction without excluding samples based on the qPCR filter. For all of the p-value calculation methods, false discovery rates were calculated from the distribution of p-values using the method of Benjamini and Hochberg [58].

Additional details and analyses using alternative statistical methods, including those that treat age and collection data as continuous variables, can be found within the “*Archive Type Full Statistical Analysis,*” “*Age Analysis,*” “*Ancestry Analysis,*” “*Collection Date Analysis,*” and “*Histological Subtype Analysis*” in [46].

## Results

### Patient sample and demographic data

Of all specimens analyzed (counting samples from the same patient more than once, n = 135), 25.0% (n = 34) were archived DNA (27 ICC samples, one vulvar cancer sample, and six prostate samples, including five cancer and one normal, as negative controls), 32.3% (n = 44) were frozen tissue (40 ICC samples and four adjacent normal cervical tissues micro-dissected from the same patient), and 42.6% (n = 57) were FFPE tissue (55 ICC samples, one mixed ICC and endometrial cancer sample, and one non-malignant vaginal sample). The mean and distribution of collection dates for FFPE tissue samples were significantly different from those of the frozen tissue samples (Mann–Whitney U test p-value = 6.4 × 10^–5^, Kolmogorov–Smirnov test p-value = 9.6 × 10^–6^). Sample information and patient demographic data are summarized in Table 1, and detailed information for each sample is provided in Additional file 2: Table S2.

**Table 1:** Summary of sample and patient characteristics

		All samples^a^	Archived DNA	Frozen tissue	FFPE tissue
Cervical samples^b^		128 (incl. 7 frozen-FFPE and 3 DNA-frozen tumor-tumor pairs)	28	44 (incl. 4 tumor-normal pairs)	56 (incl. 2 tumor-tumor pairs)
Additional samples		7	6 (prostate)	0	1 (vaginal tissue)
Samples with L1 amplicon sequencing		135	34	44	57
Number of samples with QC array data		70	26	44	0
Collection year (Mean ± SD)		1999 ± 11	Not reported	1995 ± 11	2003 ± 9
Age (Mean ± SD)		51.4 ± 14.9	Not reported	51.6 ± 15.5	50.83 ± 14.7
Histological subtype	Adenocarcinoma (AC)	25	3	9	13
	Adenosquamous Carcinoma (ASC)	6	1	0	5
	Squamous Cell Carcinoma (SSC)	87	23	27	37
	Other	3	1	1	1
	Not reported/ not applicable	14	6	7	1
Reported race^c^	Asian	11	Not reported	1	10
	Black	4		2	2
	Other	4		2	2
	White/caucasian	70		27	43
ADMIXTURE-predicted super-population assignment^d^	African (AFR)	4	0	4	Not processed
	Admixed American (AMR)	23	3	20	
	East Asian (EAS)	4	2	2	
	European (EUR)	27	16	11	
	South Asian (SAS)	0	0	0	
	AMR/EUR	7	1	6	
	AFR/EUR	1	1	0	
	SAS/EUR	1	0	1	

^a^Data shown for all samples are the sum of or summarize data only from samples for which each characteristic was reported (or estimated)

^b^Includes ICC samples, one archived DNA sample from vulval cancer, and one FFPE sample that includes a mix of ICC and endometrial cancer, as well as adjacent normal tissue

^c^Hispanic ethnicity was not reported for any samples

^d^ADMIXTURE-predicted super-population assignments were provided for samples with QC Array call rates > 75%. ADMIXTURE super-populations with contributions of > 20% to a patient’s genome are reported above

**Fig. 1:**
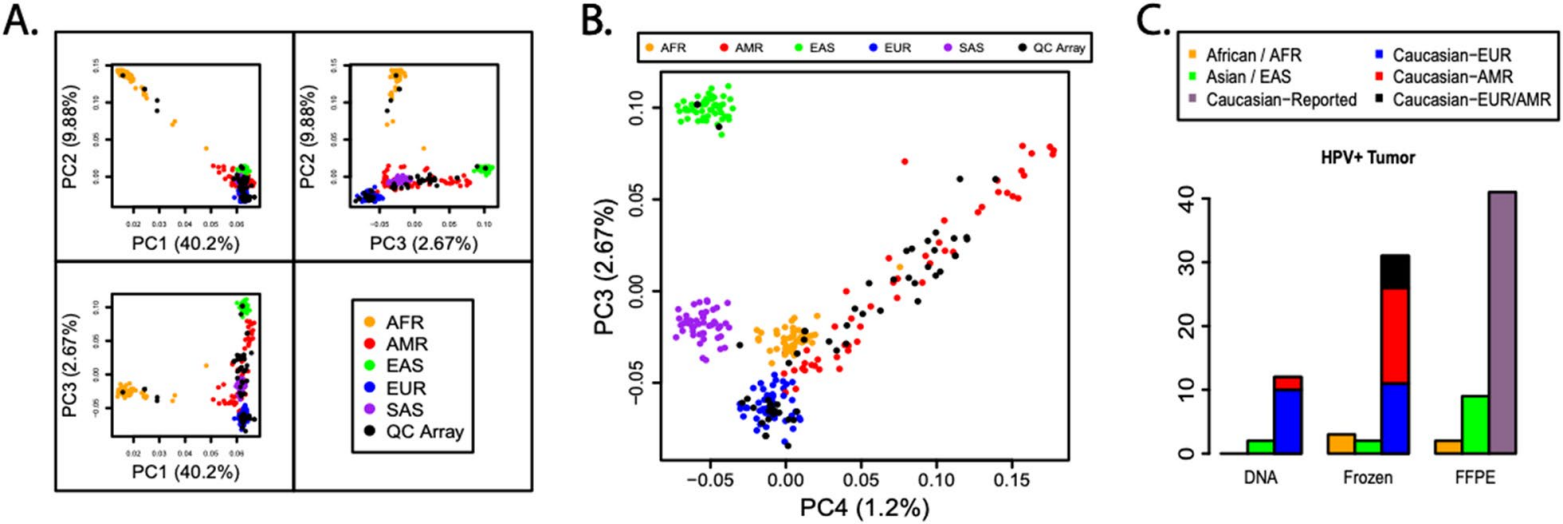
QC Array super-population assignments. **A** Super-population clusters of select 1000 Genomes reference samples and ICC samples with QC Array call rates > 85%, projected onto the first three principal components (PC1–3). 1000 Genomes individuals from AFR populations and QC Array samples from patients expected to have African ancestry were most clearly distinguished by the first two principal components. 1000 Genomes individuals from current EUR and EAS populations were separated along the third and, to a lesser extent, second principal components. **B** Similar to (**A**) but with the third principal component plotted against the fourth (PC4), demonstrating that AMR individuals become more distinct along the fourth principal component. No QC Array samples were predicted to have SAS ancestry. These principal components were not the primary method for assigning ethnicities but provide an effective way to visualize variation among samples. **C** Frequencies of reported race per archive type, as well as supervised AMR/EUR predictions for archived DNA and frozen tissues from reported “White/Caucasian” individuals. Counts are among HPV + tumor samples, counted once per patient per archive type. If race was not reported (or reported to be “Other”) or QC Array data were not available, the corresponding samples were not included in this plot

When known, the mean age of patients at sampling was 51.4 ± 14.9 years. Using Illumina Infinium QC Array data for archived DNA and frozen tissue samples, we estimated the genetic ancestry of patients and assigned them to super-populations based on a reference set of 1000 Genomes samples [56]. Super-population clusters among 1000 Genomes samples and QC Array samples with higher call rates (> 85%) are shown in Fig. 1A, B. Our sample size was limited, but our predicted super-population assignments always matched reported race: one reported Asian patient was assigned to the EAS super-population, two reported African American patients were assigned to the AFR super-population, and 27 reported Caucasian individuals were assigned to the AMR and/or EUR super-populations. Our analysis of 1000 Genomes data indicates that the AMR super-population is enriched for Hispanic individuals [56], but we do not consider AMR to be completely interchangeable with “Hispanic” in independent cohorts.

Among samples with a single super-population assignment, there were three AMR and 16 EUR archived DNA samples, compared to 20 AMR and 11 EUR frozen tissue samples. Thus, the relative frequencies of predicted AMR and EUR individuals were significantly different in frozen tissue compared to archived DNA samples (Fisher’s exact test *p*-value = 0.0011, Fig. 1C). We did not analyze FFPE samples using the QC Array.

When considering the full set of samples, there was a qualitatively greater proportion of AC and ASC (“Adeno type”) samples among archived FFPE samples than in the other archive types (Table 1 and Additional file 2: Table S2). Nevertheless, statistical significance was not achieved when comparing the relative frequencies of the Adeno types versus SSC among the three archive types (Fisher’s exact test *p*-value = 0.20). Similarly, statistical significance was not achieved when comparing the frequencies of AC alone versus SSC among the three archive types (Fisher’s exact test *p*-value = 0.37). However, focusing on the frequencies of the combined Adeno types versus SSC only in archived DNA versus FFPE tissue, the Fisher’s exact test *p*-value was 0.11.

### Variation in HPV genotype read fractions, correlations with off-target human reads, and amplified DNA concentrations by archive type

HPV genotype read fractions were calculated based on alignment to the reference genomes of 35 HPV genotypes [14, 16, 17], as well as the human genome (hg38; Additional file 1: Table S1). In six prostate tissue samples, in which HPV infection was unlikely, we measured 0.6–12.5% HPV−associated reads (with three samples having > 5% HPV−associated reads) and 84.5–98.6% off-target human reads (Additional file 2: Table S2). Frozen normal cervical tissues (adjacent to collected ICC tissues) had HPV read fractions of 53.6–96.7%, which were too high to consider these HPV− control samples. The non-malignant vaginal FFPE sample had 98.9% HPV−associated reads.

**Fig. 2:**
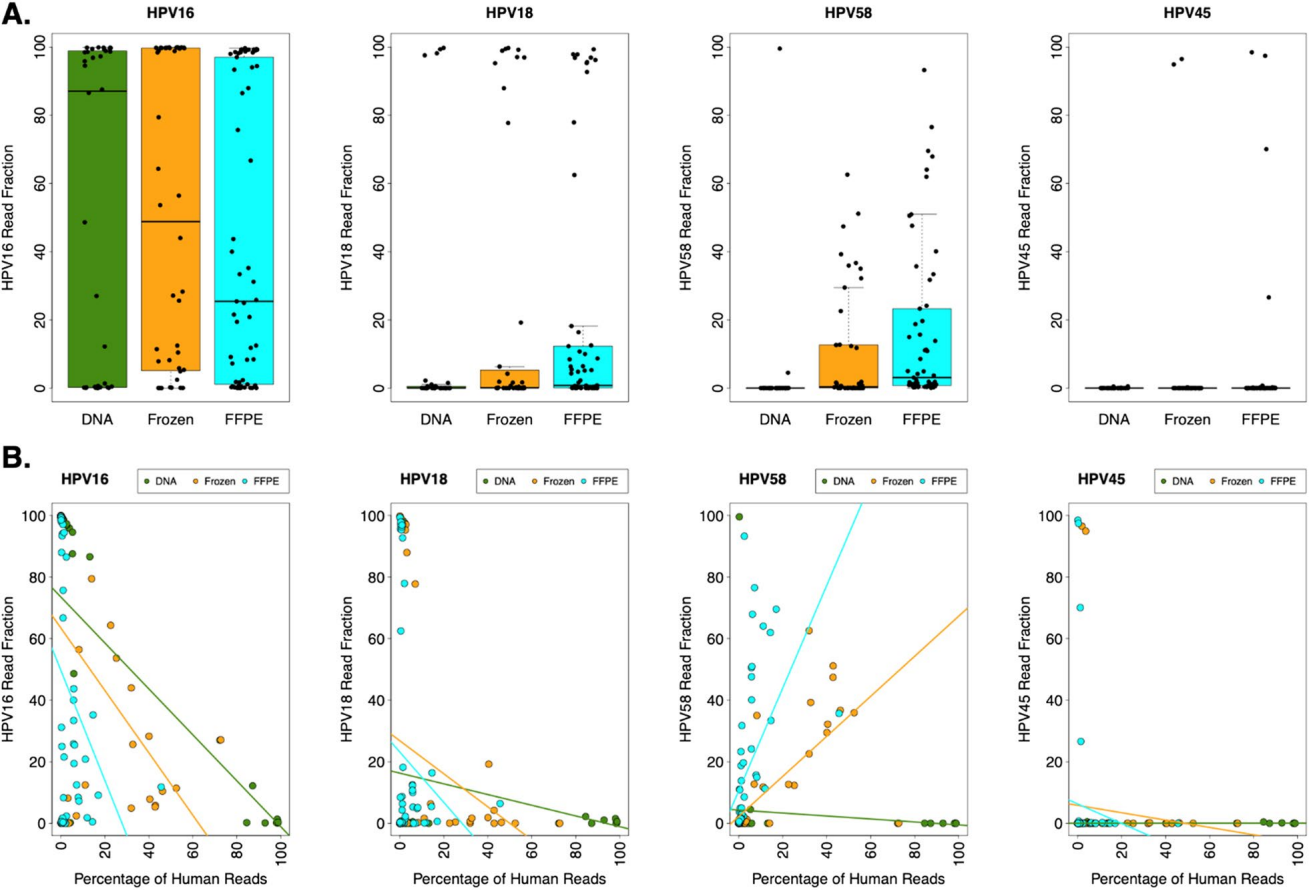
HPV58 read fractions and the percentage of off-target human reads vary by archive type. **A** Box-plots showing the read fractions of HPV16, HPV18, HPV58, and HPV45 in archived DNA, frozen tissue, and FFPE tissue samples. Although read fractions tended to be lower for HPV58 than for HPV16 and HPV18, HPV58 was detected more frequently (especially at read fractions between 20 and 80%) in FFPE tissue samples than in archived DNA and frozen tissue samples. **B** Associations between HPV16, HPV18, HPV58, and HPV45 read fractions and the percentages of off-target human reads in all samples. For HPV16 and HPV18, the HPV genotype-specific read fractions tended to be lower in samples with higher percentages of off-target human reads. In contrast, the frequency of HPV58 reads tended to be higher in frozen and FFPE tissue samples with higher percentages of human reads

Archived DNA samples from ICC had 0.9–99.9% HPV−associated reads, frozen ICC tissues had 27.2–99.9%, and FFPE tissues had 87.7–99.9%. We compared the HPV reads detected across all samples and identified HPV16, HPV18, HPV58, and HPV45 as the most prevalent genotypes. We did not detect significant differences in HPV16, HPV18, or HPV45 read fractions across archive types. However, HPV58 read fractions were higher in FFPE tissue samples than in frozen tissue and archived DNA samples, with an overall ANOVA p-value of 0.010 and an ANOVA p-value of 0.0051 for FFPE tissue versus archived DNA alone (**Fig. ****2****A**).

Interestingly, we discovered that the percentage of human reads in each sample varied by archive type (ANOVA *p*-value = 0.00012). We also observed that the median insert size of the off-target human reads was smaller for the FFPE samples than for the other archive types (overall ANOVA p-value = 9.4 × 10^–8^, see “*Median Off-Target Human Insert Size Distribution*” in [46]). Furthermore, the relationship between HPV genotype-specific read fractions and the percentage of off-target human reads in each sample varied between HPV16, HPV18, HPV58, and HPV45, as well as between archive types (Fig. 2B). Using linear regression, we detected significant negative correlations between HPV16 read fractions and the percentage of human reads for all archive types (archived DNA: r = − 0.64, *p*-value = 4.4 × 10^−5^; frozen tissue: r = − 0.43, *p*-value = 0.0039; FFPE tissue: r = − 0.29, *p*-value = 0.026). Similarly, but without reaching statistical significance, there were negative correlations between HPV18 read fractions and the percentage of human reads for all archive types (archived DNA: r = − 0.22, *p*-value = 0.21; frozen tissue, r = − 0.26, *p*-value = 0.086; FFPE tissue: r = − 0.16, *p*-value = 0.23). In contrast, HPV58 read fractions were positively correlated with the percentage of human reads in both frozen and FFPE tissue samples (frozen tissue: r = 0.71, *p*-value = 5.3 × 10^−8^; FFPE tissue: r = 0.49, *p*-value = 1.1 × 10^−4^) but not in archived DNA (r = − 0.12, *p*-value = 0.52).

To explore the potential underlying causes of these correlations, we used BLAT (the BLAST-like alignment tool) [60] to compare the most common representative sequences for each HPV genotype to the hg38 human reference genome [61]. The representative HPV16 sequence had three human BLAT hits, the HPV18 sequence had no hits, and the HPV58 sequence had six hits. More than half of the BLAT hits for HPV58 in the human genome (4 of 6) overlapped with long interspersed nuclear element (LINE) annotations, according to the RepeatMasker track of the UCSC Genome Browser [62, 63] (more information and additional analyses can be found within *“Representative Sequence Analysis”* in [46]). Considering that ten out of 14 samples with percentages of human reads between 20 and 80% had HPV58 read fractions greater than 20%, these findings suggest that homology with human genome sequences may contribute to the positive association between HPV58 reads and the percentage of human reads. Similarly, some samples with human read frequencies between 20 and 80% had HPV16 read fractions greater than 20%, which may yield a similar but a more subtle effect on HPV16 quantification. Almost no samples with human read frequencies between 20 and 80% had HPV18 read fractions greater than 20% for HPV18.

**Fig. 3:**
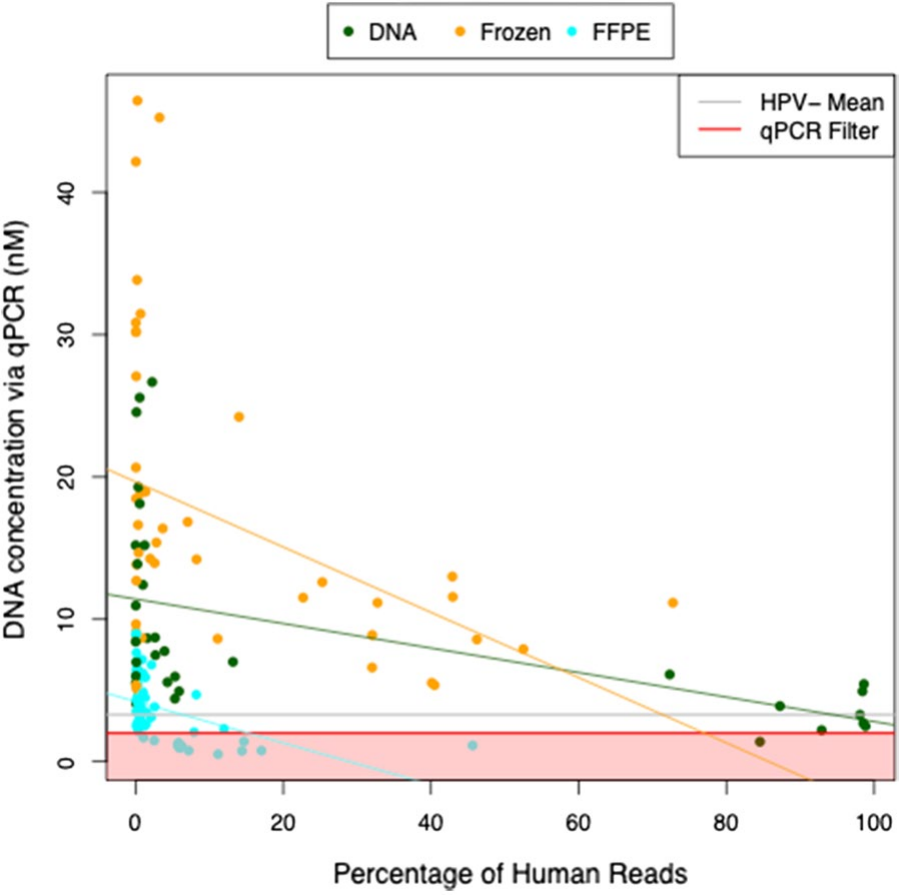
Amplified DNA concentrations vary by archive type. Concentrations of DNA amplified from each sample, quantified by qPCR after gel extraction, plotted against the percentage of off-target human reads detected by L1 amplicon sequencing. Samples are color-coded by archive type: archived DNA, frozen tissue, or FFPE tissue. The gray line indicates the average DNA concentration for HPV− archived DNA samples. Samples with DNA concentrations < 2 nM, indicated by red shading on the plot, were excluded from our HPV genotype analyses

After purifying and extracting the L1 amplicons, we used qPCR to calculate the amount of DNA amplified from each sample (Additional file 2: Table S2), which revealed significant differences between the three archive types (ANOVA *p*-value = 1.6 × 10^−14^). Furthermore, we found that the amplified DNA concentrations correlated negatively with the percentage of human reads in each sample, across archive types (r = − 0.19, linear regression *p*-value = 0.026, Fig. 3). These negative correlations were even stronger when the data was split by archive type: archived DNA (r = − 0.51, linear regression *p*-value = 0.0019), frozen tissue (r = − 0.40, linear regression *p*-value = 0.0079), and FFPE tissue (r = − 0.47, linear regression *p*-value = 0.00024). These findings indicate that amplified DNA concentrations may be a suitable quality control measure by which to exclude samples with high percentages of human reads and potentially unreliable HPV read fractions.

### HPV genotype frequencies of L1 amplicon sequences

As low DNA concentrations may yield inaccurate genotyping results, we established a 2-nM threshold to exclude samples from our analysis. This threshold was selected because it excludes the negative control with the highest percentage of HPV−associated reads (Fig. 3). Considering the HPV read fractions observed in both ICC samples and negative control prostate samples, we also established that a read fraction of at least 20% should be required to determine overall HPV + status and to assign specific HPV genotypes (see “*Effect of Human Read Threshold on Genotypes*” in [46]). The effects of adjusting this read threshold on HPV58 and co-infection assignments can be found in Additional File 3: Table S3. Subsequently, through an iterative process (Additional file 4: Table S4), we established additional criteria: overall HPV reads > 1.2 × human reads and genotype-specific HPV reads > 1.0 × human reads.

Using these criteria, the prevalence of HPV overall across ICC samples was 95%, with specific HPV genotype results similar to those previously reported (Table 2) [16, 18]. HPV16 was the most common genotype, detected in 56% of all ICC samples (68% of archived DNA, 52% of frozen tissue, and 52% of FFPE samples). HPV18 was the second most frequent, detected in 21% of all ICC samples (14% of archived DNA, 20% of frozen tissue, and 26% of FFPE tissue), followed by HPV58 and HPV45, which were both detected in 5–10% of samples, across archive types. Raw read counts and genotype assignments using alternate criteria can be accessed on GitHub [64].

**Table 2:** Summary of HPV Genotypes and Co-Infections Among ICC Patients and Samples

		Total patients	Total samples	Archived DNA samples	Frozen tissue samples	FFPE tissue samples
Number of ICC samples^a^		112	124	28	40	56
Samples passing qPCR filter		102 (91%)	110 (89%)	28 (100%)	40 (100%)	42 (75%)
HPV + patients/samples		97 (95%)	105 (95%)	25 (89%)	38 (95%)	42 (100%)
Overall “Unclear” samples		3 (3%)	3 (3%)	1 (4%)	2 (5%)	0 (0%)
HPV genotype-positive samples^b^	HPV16	59 (58%)	62 (56%)	19 (68%)	21 (53%)	22 (52%)
	HPV18	19 (19%)	23 (21%)	4 (14%)	8 (20%)	11 (26%)
	HPV31	2 (2%)	2 (2%)	0 (0%)	0 (0%)	2 (5%)
	HPV33	2 (2%)	2 (2%)	0 (0%)	0 (0%)	2 (5%)
	HPV45	6 (6%)	6 (5%)	0 (0%)	2 (5%)	4 (10%)
	HPV58	8 (8%)	8 (7%)	1 (4%)	4 (10%)	3 (7%)
	HPV59	2 (2%)	3 (3%)	2 (7%)	1 (3%)	0 (0%)
	HPV67	1 (1%)	1 (1%)	0 (0%)	0 (0%)	1 (2%)
	HPV73	2 (2%)	2 (2%)	0 (0%)	0 (0%)	2 (5%)
	Unclear^c^	2 (2%)	2 (2%)	0 (0%)	2 (5%)	0 (0%)
HPV genotypes per sample	1		100 (91%)	24 (86%)	38 (95%)	38 (90%)
	2		4 (4%)	1 (4%)	0 (0%)	3 (7%)
	3		1 (1%)	0 (0%)	0 (0%)	1 (2%)
	4		0 (0%)	0 (0%)	0 (0%)	0 (0%)

^a^Includes one archived DNA sample extracted from vulval cancer, as well as one FFPE sample that includes a mix of ICC and endometrial cancer; excludes negative control prostate samples, ICC-adjacent normal tissues, and non-malignant vaginal sample

^b^Because of co-infections, one sample can contribute to the counts of multiple HPV genotypes

^c^Samples with an “unclear” genotype meet the read requirement for overall HPV reads but not for any particular HPV genotype. For patients, only one assignment in one sample was required to be designated positive for a given HPV genotype (i.e., “clear”)

### *Limitations of GP5* + */GP6* + *primer amplification*

It should be noted that some HPV types are not well-amplified using the GP5 + /6 + primers. For example, the HPV52 reference genome has five sequence differences compared to the forward primer and three sequence differences compared to the reverse primer [65]. If we use BWA-MEM to quantify off-target human reads, given the small fraction of read counts for HPV52, it is hard to distinguish signal from noise (Additional file 5: Table S5). If we use Bowtie1 [66] to align reads, then we lose some human read alignments. However, there was one FFPE sample (S16142.01.12) with very low read counts for HPV52 (< 0.1% read fraction) that passed a visual inspection for alignment (see “*Bowtie1 comparison / HPV52 low coverage*” in [46]). Nevertheless, as a rule, we did not assign specific HPV genotypes to samples with read fractions below 1% using the GP5 + /6 + primer set. This does not mean that there were no reads originating from those genotypes. However, systematically distinguishing signal from background noise is difficult, if not impossible, using read counts alone.

### Previous genotype assignments in archived DNA samples

Because the archived DNA samples had been previously assigned HPV genotypes based on RFLP analysis (Additional file 2: Table S2), we sought to identify concordance between the previous genotypes and the genotypes assigned based on our L1 amplicon sequencing and genotyping criteria. We observed concordant HPV genotype results for 17/18 HPV16 + samples, 4/4 HPV18 + samples, and 0/1 HPV45 + samples, as well as concordant HPV− results for 8/9 samples (including six negative control prostate samples). Of the two samples that were assigned a genotype of “other” (i.e., a rare specific genotype) based on RFLP analysis, one (E772.25) was re-assigned as HPV16 + based on 87.5% HPV16 reads, and the other (E373.65) was re-assigned as HPV59 + based on 94.5% HPV59 reads. We confirmed that one sample previously identified as HPV45 + (E540.20) was HPV + , but we re-assigned it as HPV58 + based on 99.5% HPV58 reads. One sample previously identified as HPV16 + (E555.07) contained 27.0% HPV16 reads but also 72.3% off-target human reads, so we labeled it as having an “unclear” genotype rather than definitively HPV16 + or HPV−.

Two ICC samples that were concordantly identified as HPV− had HPV frequencies < 15%. A third sample previously identified as HPV− (E741.18) was re-assigned as HPV + with a mix of HPV16 reads (48.6%) and HPV59 reads (44.8%). This re-assignment may reflect a co-infection; however, it may be due to a low percentage of tumor cells in the sample. For comparison, the qPCR concentration for E741.18 (4.9 nM) was similar to that of two HPV− ICC samples (3.3 nM and 2.5 nM). The negative control prostate samples, which had HPV read fractions up to 12.45% but were considered HPV− based on our genotyping criteria, had qPCR concentrations ranging from 1.4 to 5.4 nM. In contrast, one HPV18 + ICC sample had an intermediate concentration of 4.1 nM, and the vulvar cancer sample (E772.25), which had a concentration of 4.4 nM, was re-assigned HPV16. Therefore, it is not possible to definitively determine the HPV status of this sample given the data available.

### Effects of excluding FFPE samples based on amplified DNA concentration

**Fig. 4:**
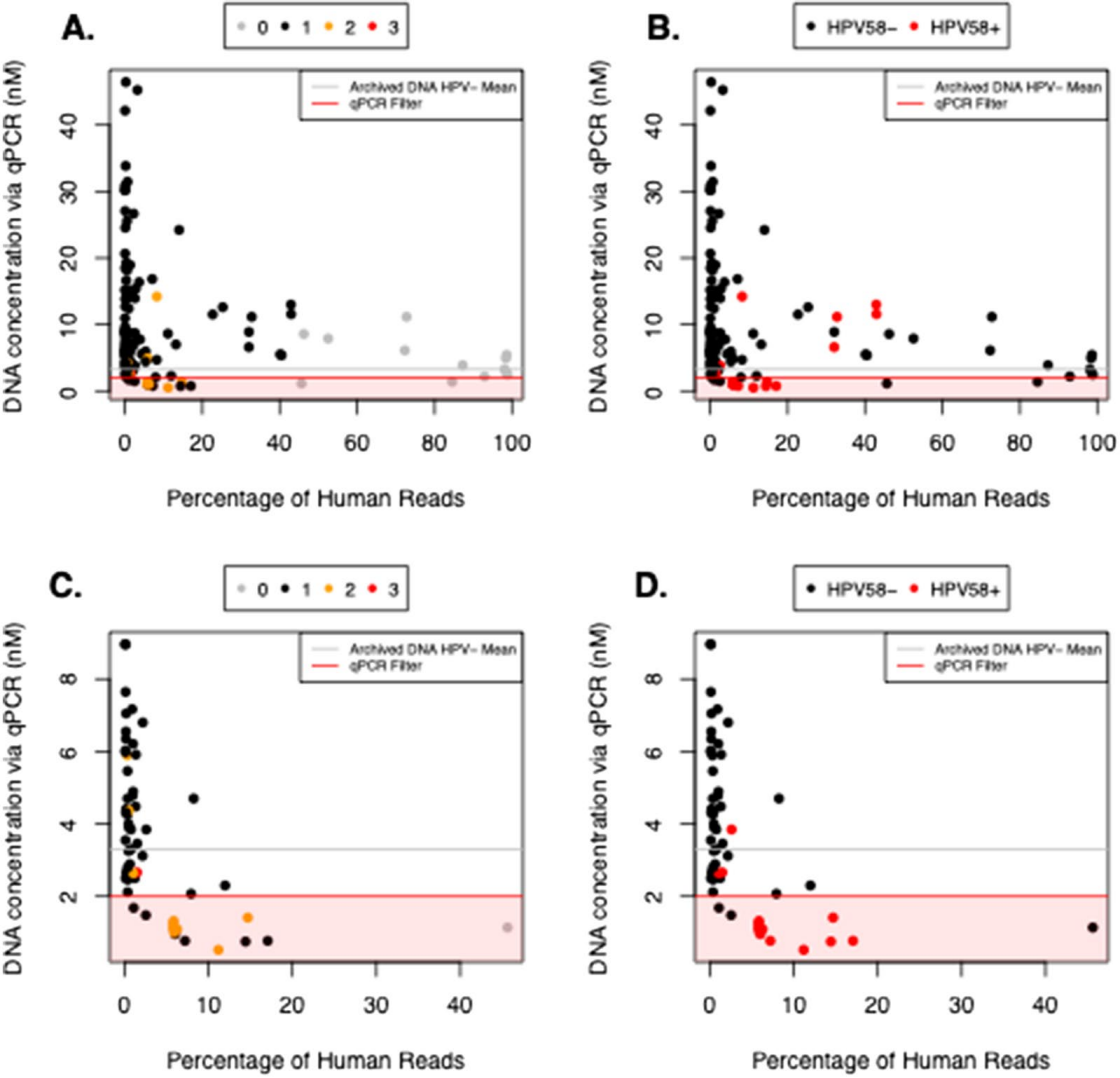
Excluding FFPE samples based on amplified DNA concentrations likely reduces false-positive HPV58 genotype and HPV co-infection assignments. Concentrations of DNA amplified from all samples (**A–B**), as shown in Fig. 3, or FFPE samples only (**C–D**). Samples are color-coded by the number of HPV co-infections detected (**A** and **C**) or their assigned HPV58 genotype status (**B** and **D**). The gray lines indicate the average DNA concentration for HPV− archived DNA samples. The 2-nM threshold used to filter samples is indicated by red shading

To assess the broader effects of excluding samples based on our amplified DNA concentration threshold (“qPCR filter”), we analyzed the frequency of HPV genotypes and co-infections using the full set of data, including samples with amplified DNA concentrations < 2 nM (Fig. 4). Notably, without excluding any samples, we would have detected considerably more putative co-infections in the FFPE samples, potentially indicating contamination. We would have also detected several more HPV58 + samples, which are typically rarer than HPV16 + and HPV18 + samples [16, 18, 19].

Furthermore, we compared the number of HPV58 reads in replicate tumor samples from the same patient and found that, without excluding any samples, the coefficient of correlation between HPV58 read fractions for paired HPV58 + samples was not ideal (r = 0.77, Additional file 6: Fig. S1). Using the same set of unfiltered samples, the coefficients of correlation for HPV16 read fractions between HPV16 + tumor pairs and HPV18 read fractions between HPV18 + tumor pairs were approximately 0.97 and 1.00, respectively. When the qPCR filter was applied, all HPV58 + pairs were eliminated, so the data were insufficient to meaningfully assess how the correlation between paired HPV58 samples changed based on our revised genotyping criteria. In addition to reducing potentially false-positive HPV58 assignments, excluding data with low amplified DNA concentrations may have increased the robustness of our HPV16 assignments, as the coefficient of correlation between HPV16 read fractions increased to approximately 1.00 for paired HPV16 + samples after the filter was applied (Additional file 6: Fig. S1).

### HPV genotype frequency variation by archive type, histological subtype, collection date, patient age, and super-population

Given that HPV58 read fractions and the correlations of HPV16, HPV18, and HPV58 read fractions with the percentage of off-target human reads varied by archive type, we used Fisher’s exact test to compare the frequencies of assigned HPV genotypes across archive types. Although the read fraction of HPV58 varied by archive type when no filters were applied, after excluding samples with low amplified DNA concentrations and establishing read threshold requirements for genotyping, there were no significant effects of archive type on the detection of HPV58 or any other HPV genotypes.

There were significant differences in the frequencies of HPV16 and HPV18 based on the histological subtype of the samples. Namely, HPV16 frequencies were higher among SCC samples than in AC and ASC samples, and HPV18 frequencies were higher in the combined group of Adeno type samples than in SCC samples (“*Histological Subtype Analysis*” in [46]). For both of these HPV genotypes, differences in frequency based on histological subtype were most significant in the archived DNA samples and least significant in the FFPE tissue samples. None of the other HPV genotypes had statistically significant differences in frequency based on histological subtype in our set of samples.

Using Fisher’s exact test, we did not detect any differences in HPV genotype frequencies based on sample collection date; however, we did not have collection dates for any archived DNA samples. We also failed to detect differences in HPV genotype frequencies based on patient age or between AMR and EUR individuals using Fisher’s exact test, which may similarly be due to small sample sizes rather than a true lack of effect. As we show in “*Low Frequency Sample Size Calculation*” in [46], it would be beneficial to have a total sample size closer to 200 to detect a 15% versus 5% difference in genotype frequency and a sample size closer to 500 to detect a 10% versus 5% difference. The total sample size for 2-group comparisons in this study was fewer than 95.

## Discussion

In this pilot study, we showed that high-throughput sequencing of the L1 amplicon can be used to assign HPV genotypes in archived ICC samples. We also demonstrated that a low-density genotyping array (Illumina Infinium QC Array) can be used to generate population-level ancestry estimates and match samples obtained from the same patient. Intriguingly, we observed that specific HPV genotype frequencies varied by archive type and based on the percentage of off-target human reads in each sample. Moreover, we found that the amount of DNA amplified from each sample varied by archive type and correlated negatively with the percentage of human reads in each sample. We demonstrated that these experimental characteristics considerably influenced the frequency of HPV58 detection in particular.

Overall, we obtained HPV genotype frequencies consistent with those reported by others [19]. In this study, HPV16 and HPV18 were most prevalent, followed by several other genotypes detected in 10% of samples or fewer, depending on archive type (Table 2). In archived DNA and frozen tissue samples, the frequency of HPV16 was significantly higher for SCC than for the Adeno types, whereas the frequency of HPV18 was significantly higher for the Adeno types than SCC (“*Histological Subtype Analysis*” in [46]). These trends have been previously reported [19, 22, 23]; however, these differences were not observed in the FFPE samples, for which we had the largest sample size and the most recent mean collection date. This discrepancy may have occurred because storage conditions were different for the FFPE samples (room temperature versus cold temperature), and there may be disadvantages to using DNA extracted from FFPE samples stored at room temperature (as our samples were) relative to cooler temperatures [67, 68]. In addition, a previous study on the extraction of HPV DNA concluded that the extraction protocol used for FFPE samples can significantly affect the results, and different HPV primers may be preferable to the GP5 + /6 + primers used in this study [69]. Moreover, in this study, the FFPE samples had off-target human reads with noticeably smaller fragment sizes than the other archive types (see “*Median Off-Target Human Insert Size Distribution*” in [46]). Other notable observations from our FFPE samples include: removal of several samples due to low amplified DNA concentrations (not observed for DNA or frozen samples; Fig. 3) and a higher fraction of HPV58 reads (Fig. 2A). These findings indicate that experimental factors must be carefully considered when interpreting data on the prevalence of HPV genotypes in FFPE samples. The replication of these findings in a study where archive types are interspersed and randomized across sequencing batches would be ideal. In addition to histological subtype, ICC disease stage (e.g., International Federation of Gynecology and Obstetrics [FIGO] staging) may also have an impact on the HPV genotype distribution in ICC patients; although we did not have access to staging information in our datasets, we strongly recommend that such an analysis be performed in future studies.

We observed a positive correlation between HPV58 read fractions and the percentage of off-target human reads in frozen and FFPE samples (Fig. 2B). Therefore, we carefully analyzed and established HPV genotyping criteria that required overall HPV read fractions > 1.2 × human reads and specific HPV genotype reads > 1.0 × human reads (Additional File 4: Table S4). Our efforts to establish robust HPV genotyping criteria revealed that computational parameters can also dramatically affect HPV genotype frequencies. For example, we found that the number of HPV58 + FFPE samples could vary by twofold when the read thresholds were adjusted (Additional File 3: Table S3), thus highlighting the importance of transparency in reporting HPV genotyping results, as well as providing raw data for future meta-analyses.

Because low amplified DNA concentrations may yield artificially high HPV genotype read fractions, we excluded HPV + or “unclear” samples with amplified DNA concentrations lower than 2 nM. When comparing HPV16 and HPV18 read fractions in HPV16 + and HPV18 + replicate tumor-tumor pairs, respectively, we observed high correlation coefficients, before and after filtering samples based on amplified DNA concentrations and assigning genotypes using our established criteria. In fact, the correlation coefficient for HPV16 was slightly higher after removing samples with low amplified DNA concentrations. In contrast, HPV58 + tumor pairs had poorly correlated HPV58 read fractions and could no longer be defined once the qPCR filter was applied. Application of the qPCR filter also resulted in statistically similar HPV58 genotype frequencies across archive types, including FFPE tissues (Fig. 4; Table 2). These findings further confirmed the importance of establishing rational criteria for assigning HPV genotypes based on amplicon sequencing. Although our results for archived DNA samples were mostly concordant with their previous HPV genotypes assigned by RFLP analysis, it remains essential that future studies independently validate the presence of HPV58 using whole-genome sequencing or independent markers (such as the HPV58-specific primer set from Hu et al. [70]) and validate our methods in independent cohorts.

It may also be necessary to establish different criteria for each archive type, as well as for specific HPV genotypes. Additionally, we recommend including sufficient controls in every batch. For example, one study that reported relatively high rates of HPV58 + samples (> 20%) also reported that 24.69% of their healthy controls were HPV58 + [71]. Other quality control measures that could be used in the future include alternating HPV + and HPV− samples in adjacent lanes of each gel [72]. Moreover, we used single-barcode libraries in this study, which we expect to show noticeably more “barcode hopping” than dual-barcode libraries [73, 74]. Therefore, we cannot confidently conclude that the high frequency of HPV58 + FFPE samples detected using a lower read threshold (Additional file 3: Table S3) was or was not a biologically meaningful finding. For example, it remains possible that the relatively high HPV58 read frequency in FFPE samples was due, in part, to differences in the racial/ethnic distribution of the patients from whom the samples were collected and/or a change in the population frequency of HPV58 over time.

Many datasets, including our own (Table 1), lack reported race/ethnicity data, potentially hindering the discovery of critical factors that may influence the distribution and eventual detection of specific HPV genotypes in vulnerable populations. To overcome this limitation, we used QC Array probes to stratify our DNA and frozen tissue samples into super-populations (arguably similar to ethnic groups) using the program ADMIXTURE and a bootstrap simulation trained and validated using data from the 1000 Genomes project. We confirmed that super-population clustering could be observed using our QC Array analysis methods (Fig. 1), and our predicted super-population assignments always matched reported race. Thus, although we were unable to determine the impact of race/ethnicity on HPV58 frequency, as the FFPE samples were not processed using the QC Array, we demonstrated that these methods can be used to confirm reported race/ethnicity data and to address insufficient race/ethnicity data in other datasets. Future studies to validate our HPV58 genotyping criteria and to test the effects of demographic and biological factors on HPV genotypes are warranted. Moreover, it is critical to acknowledge that many characteristics of the patient population in any given geographic area, including the racial/ethnic distribution, have likely shifted over time. Therefore, if any biologically meaningful findings are obtained using archived samples, it will be essential to validate results using fresh samples from the current population to determine relevance to patients in the same region today.

## Conclusions

In this study, we evaluated L1 amplicon sequencing data from archived DNA, FFPE tissue, and frozen tissue samples. Our analysis revealed that the percentage of human reads and the concentration of L1 DNA amplified from each sample are critical factors to consider when evaluating HPV genotype frequencies. After accounting for the percent of human reads in each sample and excluding samples with especially low levels of amplified DNA, our sample sizes were not robust enough to evaluate the effects of patient age and race/ethnicity or sample collection date on assigned HPV genotype frequency; however, among our samples, the read fraction of HPV58 was significantly greater in FFPE samples than in archived DNA or frozen tissue samples. We also detected higher frequencies of HPV16 in SCC and higher levels of HPV18 in AC and ASC in archived DNA and frozen tissue samples but not FFPE samples. While further studies are required to determine the underlying causes of these observations, these findings suggest that experimental and computational processing methods can influence the detection of oncogenic HPV genotypes. Considering the impact of experimental factors and computational parameters on our HPV genotyping results, we recommend that any raw data used for HPV genotyping be made available so that future meta-analyses can evaluate whether the original genotyping strategies were appropriate.

### Supplementary Information

**Additional file 1.** Reference sequences used for HPV genotyping.

**Additional file 2.** Detailed data for each sample, including archive type, tissue type, histological subtype, collection year, details for partial consent validation ("Consent Notes"), patient age at sample collection, reported race, percentage of off-target human reads, overall HPV status (“unclear” indicates a high fraction of human reads, “qPCR filter” indicates a low amplified DNA concentration), HPV genotype assignments (using a 20% read fraction threshold and other standard criteria defined in the Methods), percentages of specific HPV genotype reads, previous archived DNA genotyping results (based on RFLP analysis), amplified DNA concentrations (quantified by qPCR after gel extraction), median and maximum sizes among human-aligned reads (calculated from paired-end reads using Picard), paired sample IDs (based on provided annotations, QC Array IBD calculations, or both), QC Array call rate (a QC measure for the sample), primary super-population assignments (based on >50% estimated contributions, using ADMIXTURE), mixed super-population assignments (based on >20% estimated contributions, ADMIXTURE), distance-based bootstrap super-population assignments (with confidence >95%), HPV L1 amplicon sequencing barcode, run information (run number: flowcell ID: lane number), and total HPV L1 amplicon sequencing reads (combined between lanes).

**Additional file 3.** Effect of varying read thresholds for detecting HPV58 and HPV co-infections.

**Additional file 4.** Effect of varying off-target human read thresholds on HPV genotype.

**Additional file 5.** Adjusted read counts from BWA-MEM alignment to a joint reference set, including the human genome (hg38) and a 35-HPV genotype reference set.

**Additional file 6.** Effect of the qPCR filter on HPV genotype read frequencies in paired tumor samples. **(A)** Consistency in the percentages of HPV16, HPV18, and HPV58 reads was evaluated using three types of tumor-tumor pairs: pairs of FFPE tissue samples from the same patient, as reported in patient records (“FFPE:Both”); pairs of frozen and FFPE tissue samples from the same patient, as reported in patient records (“Mixed:Reported”); and pairs of archived DNA and frozen tissue samples, matched via QC Array data (not reported in sample records, “Mixed:QCarray”). Correlations between the read frequencies for each pair were lower for HPV58 than for HPV16 and HPV18. **(B)** Same as **(A)** but excluding samples that did not pass the qPCR filter (amplified DNA concentrations < 2 nM).

## Data Availability

HPV L1 DNA sequencing reads are available from the NCBI Sequence Read Archive (SRA) in PRJNA420360, with human reads from the trimmed alignment filtered before the SRA upload. Because the samples contain identifying genetic information and we lacked explicit consent to make this data public, QC Array sequence data are not available from the GEO database. We provide code for genotyping in this GitHub repository: https://github.com/cwarden45/HPV_type_paper-archived_samples. Please note that total read counts are needed to make the genotyping assignments, but the SRA dataset provides only filtered human reads (from our joint alignment of adapter-trimmed reads against the human + 35 HPV reference set, prior to data deposit). Code to reproduce the figures and tables is available at https://github.com/cwarden45/HPV_type_paper-archived_samples/tree/master/Downstream_R_Code. The full L1 amplicon reads (with human reads) and QC Array SNP chip data are available from the corresponding author upon reasonable request.

## Abbreviations

AC: Adenocarcinoma
AFR: African super-population
AMR: Admixed American super-population
ASC: Adenosquamous carcinoma
BLAT: BLAST-like alignment tool
CIN: Cervical intraepithelial neoplasia
EAS: East Asian super-population
EUR: European super-population
FFPE: Formalin-fixed paraffin-embedded
HPV: Human papillomavirus
IBD: Identity-by-descent
ICC: Invasive cervical cancer
LINE: Long interspersed nuclear element
qPCR: Real-time quantitative PCR
RFLP: Restriction fragment length polymorphism
SAS: South Asian super-population
SCC: Squamous cell carcinoma
