# Correction: HPV genotyping by L1 amplicon sequencing of archived invasive cervical cancer samples: a pilot study

**DOI:** 10.1186/s13027-024-00589-0

**Published:** 2024-06-19

**Authors:** Charles D. Warden, Preetam Cholli, Hanjun Qin, Chao Guo, Yafan Wang, Chetan Kancharla, Angelique M. Russell, Sylvana Salvatierra, Lorraine Z. Mutsvunguma, Kerin K. Higa, Xiwei Wu, Sharon Wilczynski, Raju Pillai, Javier Gordon Ogembo

**Affiliations:** 1 Integrative Genomics Core, City of Hope National Medical Center, Duarte, CA 91010 USA; 2 Department of Medicine, Vanderbilt University Medical Center, Nashville, TN 37212 USA; 3 Molecular Pathology Core, City of Hope National Medical Center, Duarte, CA 91010 USA; 4 Research Informatics, City of Hope National Medical Center, Duarte, CA 91010 USA; 5 Clinical Informatics, City of Hope National Medical Center, Duarte, CA 91010 USA; 6 Biorepository, City of Hope National Medical Center, Duarte, CA 91010 USA; 7 Department of Immuno‑Oncology, City of Hope National Medical Center, Duarte, CA 91010 USA; 8 Office of Faculty and Institutional Support, City of Hope National Medical Center, Duarte, CA 91010 USA; 9 Department of Pathology, City of Hope National Medical Center, Duarte, CA 91010 USA

**Correction: Infectious Agents and Cancer (2022) 17:44** 10.1186/s13027-022-00456-w

In this article, the authors identified the following errors:

1. Reference list: Original reference 17 (Van Doorslaer et al., 2013) needed to be re-numbered to reference 59 (Van Doorslaer and Burk, 2010), and vice versa.
2. ‘HPV genotype reference set development’ subsection: In the sentence beginning “Our final reference set of 35 HPV genotypes..”, the citation to references [14–17] needed to be corrected to “[14, 16, 17].
3. ‘HPV genotype reference set development’ subsection: In the sentence beginning “We also used the PaVE database..”, the citation to reference [17] needed to be corrected to [59].
4. In the ‘Variation in HPV genotype read fractions, correlations with off‑target human reads, and amplified DNA concentrations by archive type’ subsection title, the abbreviation “dna” needed to be corrected to “DNA”
5. ‘Variation in HPV genotype read fractions, correlations with off‑target human reads, and amplified DNA concentrations by archive type’ subsection: In the sentence beginning “HPV genotype read fractions were calculated..”, the citation to references [14, 16, 59] needed to be corrected to “[14, 16, 17].
6. File for Additional file 1: This file required multiple corrections and the required corrections are marked in red in the Additional file 1 attached to this Correction article.
7. File for Additional file 2: In this file, a minor typo in the sheet name needed to be corrected.
8. File for Additional file 3: In this file, the citation to reference 44 in the footnotes beginning “*The effect of adjusting the read..*” and “*Excluding samples with low amplified..*” was incorrect and needed to be removed, because the URL to the source was included already.
9. File for Additional file 4: In this file, the citation to reference 44 in the footnote beginning “The combination of the 20% read..” was incorrect and needed to be removed, because the URL to the source was included already.

The original article and Additional files 1–4 have been corrected.

### Supplementary Information

**Additional file 1.**

