# Supplementary material for: HPV genotyping by L1 amplicon sequencing of archived invasive cervical cancer samples: a pilot study
Source: Infect Agent Cancer. 2022 Aug 9;17:44. doi: 10.1186/s13027-022-00456-w (PMC9361560; doi:10.1186/s13027-022-00456-w)
Supplement: Supplementary file 3 — Additional file 3. Effect of varying read thresholds for detecting HPV58 and HPV co-infections. [file 13027_2022_456_MOESM3_ESM.docx]

**Table S3: Effect of Varying Read Thresholds for Assigning HPV58 Genotype Status and Detecting HPV Co-Infections**

**A. Number of samples positive for HPV58, depending on read thresholds**

| **Read Threshold^a^** | **Archived DNA**  **(n=28^b^)** | **Frozen Tissue**  **(n=40^c^)** | **FFPE Tissue**  **(n=56^d^)** |
| --- | --- | --- | --- |
| **HPV58 Detected**  **(>5% Reads)** | 1 (3.6%) | 6 (15.0%) | 22 (39.3%) |
| **HPV58 Alternative Detection Status**  **(>20% Reads)** | 1 (3.6%) | 4 (10.0%) | 14 (25.0%) |
| **HPV58 Plurality**  **(Most Abundant HPV-Type)** | 1 (3.6%) | 4 (10.0%) | 10 (17.9%) |
| **HPV58 Dominant / Majority**  **(>50% Reads)** | 1 (3.6%) | 2 (5.0%) | 8 (14.3%) |

^a^All strategies for HPV58 detection require that total HPV reads > 1.2x human reads and HPV58 reads > 1.0x human reads.

^b^Excluding archived DNA samples from the 6 negative control prostate samples.

^c^Excluding frozen adjacent normal samples.

^d^Excluding non-malignant vagina sample.

*The effect of adjusting the read threshold is reduced when samples with low amplified DNA concentrations are excluded, see the “Effect of qPCR Flag on HPV58 Genotypes” in:* <https://github.com/cwarden45/HPV_genotype_paper-archived_samples/tree/master/Downstream_R_Code/Extra_Analysis/Effect_of_qPCR_Flag_on_HPV58_Genotypes>

**B. Number of samples with HPV co-infections, depending on read thresholds**

| **Read Threshold** | **Co-Infection Category** | **Archived DNA** | **Frozen Tissue** | **FFPE Tissue** |
| --- | --- | --- | --- | --- |
| **>5% Reads** | **2+ HPV Co-Infection** | 1 (3.6%) | 2 (5.0%) | 21 (37.6%) |
|  | **3+ HPV Co-Infection** | 0 (0.0%) | 1 (2.5%) | 12 (21.5%) |
| **>20% Reads** | **2+ HPV Co-Infection** | 1 (3.6%) | 0 (0.0%) | 11 (17.9%) |
|  | **3+ HPV Co-Infection** | 0 (0.0%) | 0 (0.0%) | 1 (1.8%) |
| **>50% Reads** | **HPV- / Unclear Samples**  **[No Majority Genotype]** | 4 (14.3%) | 7 (17.5%) | 6 (10.7%) |

*Excluding samples with low amplified DNA concentrations further reduces the number of co-infections detected in FFPE samples, see “Effect of qPCR Flag on Co-Infections” in*: <https://github.com/cwarden45/HPV_genotype_paper-archived_samples/tree/master/Downstream_R_Code/Extra_Analysis/Effect_of_qPCR_Flag_on_Co-Infections>
