# Supplementary material for: HPV genotyping by L1 amplicon sequencing of archived invasive cervical cancer samples: a pilot study
Source: Infect Agent Cancer. 2022 Aug 9;17:44. doi: 10.1186/s13027-022-00456-w (PMC9361560; doi:10.1186/s13027-022-00456-w)
Supplement: Supplementary file 4 — Additional file 4. Effect of varying off-target human read thresholds on HPV genotype. [file 13027_2022_456_MOESM4_ESM.docx]

**Table S4: Effect of Varying Off-Target Human Read Thresholds on HPV Genotype Assignments**

1. **Effect for all data (without qPCR filter)**

|  | | **Parameters for Assigning HPV Genotypes**^a^ | | | |
| --- | --- | --- | --- | --- | --- |
| **Overall HPV Threshold** | | **> 1.5x Human** | **> 1.2x Human** | **> 1.0x Human** | **> 0.8x Human** |
| **HPV Genotype Threshold** | | **> 1.2x Human** | **> 1.0x Human** | **> 0.8x Human** | **> 0.6x Human** |
| **Samples Positive for HPV Genotypes** | HPV16 | 74 | 74 | 74 | 76 |
|  | HPV18 | 24 | 24 | 24 | 24 |
|  | HPV58 | 17 | 20 | 20 | 26 |
| **HPV58/HPV18 Ratio^b^** | Archived DNA | 0.25 (1 HPV58) | 0.25 (1 HPV58) | 0.25 (1 HPV58) | 0.25 (1 HPV58) |
|  | Frozen Tissue | 0.22 (2 HPV58) | 0.56 (5 HPV58) | 0.56 (5 HPV58) | 1.11 (10 HPV58) |
|  | FFPE Tissue | 1.27 (14 HPV58) | 1.27 (14 HPV58) | 1.27 (14 HPV58) | 1.36 (15 HPV58) |
| **Samples with “Unclear” Genotype Assignments^c^** | | **Overall:** 1 DNA, 7 frozen, 1 FFPE  **Genotype-specific:** 1 frozen | **Overall:** 1 DNA, 3 frozen, 1 FFPE  **Genotype-specific:** 2 frozen | **Overall:** 1 DNA, 2 frozen  **Genotype-specific:** 3 frozen, 1 FFPE | **Overall:** 1 DNA, 1 frozen  **Genotype-specific:** [none] |

1. **Effect excluding data with low amplified DNA concentration (*with* qPCR filter)**

|  | | **Parameters for Assigning HPV Genotypes**^a^ | | | |
| --- | --- | --- | --- | --- | --- |
| **Overall HPV Threshold** | | **> 1.5x Human** | **> 1.2x Human** | **> 1.0x Human** | **> 0.8x Human** |
| **HPV Genotype Threshold** | | **> 1.2x Human** | **> 1.0x Human** | **> 0.8x Human** | **> 0.6x Human** |
| **Samples Positive for HPV Genotypes** | HPV16 | 65 | 65 | 65 | 67 |
|  | HPV18 | 24 | 24 | 24 | 24 |
|  | HPV58 | 6 | 9 | 9 | 14 |
| **HPV58/HPV18 Ratio^b^** | Archived DNA | 0.25 (1 HPV58) | 0.25 (1 HPV58) | 0.25 (1 HPV58) | 0.25 (1 HPV58) |
|  | Frozen Tissue | 0.22 (2 HPV58) | 0.56 (5 HPV58) | 0.56 (5 HPV58) | 1.11 (10 HPV58) |
|  | FFPE Tissue | 0.27 (3 HPV58) | 0.27 (3 HPV58) | 0.27 (3 HPV58) | 0.27 (3 HPV58) |
| **Samples with “Unclear” Genotype Assignments^c^** | | **Overall:** 1 DNA, 7 frozen  **Genotype-specific:** 1 frozen | **Overall:** 1 DNA, 3 frozen  **Genotype-specific:** 2 frozen | **Overall:** 1 DNA, 2 frozen  **Genotype-specific:** 3 frozen | **Overall:** 1 DNA, 1 frozen  **Genotype-specific:** [none] |

^a^All strategies require at least 20% HPV reads overall and for specific genotypes. See **Table S3** for the effects of varying the read threshold.

**^b^**Given that 1) the numbers of HPV18+ and HPV58+ samples (including co-infections) are similar, 2) there is more variation among HPV18 sequences than among HPV58 sequences, and 3) off-target human reads positively correlate with HPV58 reads, we believe that a high HPV58/HPV18 ratio likely indicates a relatively high false positive rate for HPV58 assignments.

**^c^**Samples with an “unclear” overall HPV genotype have >20% HPV reads but cannot be assigned a specific HPV genotype due to a high percentage of human reads. Samples with an “unclear” specific genotype meet the read requirement for assignment as overall HPV+ but do not meet the read requirement for any specific HPV genotype.

The combination of the 20% read fraction and qPCR filter produces the most consistent results. For additional analyses, see *“Effect of Human Read Threshold on Genotypes”* in : <https://github.com/cwarden45/HPV_genotype_paper-archived_samples/tree/master/Downstream_R_Code/Extra_Analysis/Effect_of_Human_Read_Threshold_on_Genotypes>
