# Supplementary material for: HPV genotyping by L1 amplicon sequencing of archived invasive cervical cancer samples: a pilot study
Source: Infect Agent Cancer. 2022 Aug 9;17:44. doi: 10.1186/s13027-022-00456-w (PMC9361560; doi:10.1186/s13027-022-00456-w)
Supplement: Supplementary file 6 — Additional file 6. Effect of the qPCR filter on HPV genotype read frequencies in paired tumor samples. (A) Consistency in the percentages of HPV16, HPV18, and HPV58 reads was evaluated using three types of tumor-tumor pairs: pairs of FFPE tissue samples from the same patient, as reported in patient records (“FFPE:Both”); pairs of frozen and FFPE tissue samples from the same patient, as reported in patient records (“Mixed:Reported”); and pairs of archived DNA and frozen tissue samples, matched via QC Array data (not reported in sample records, “Mixed:QCarray”). Correlations between the read frequencies for each pair were lower for HPV58 than for HPV16 and HPV18. (B) Same as (A) but excluding samples that did not pass the qPCR filter (amplified DNA concentrations < 2 nM). [file 13027_2022_456_MOESM6_ESM.pdf]

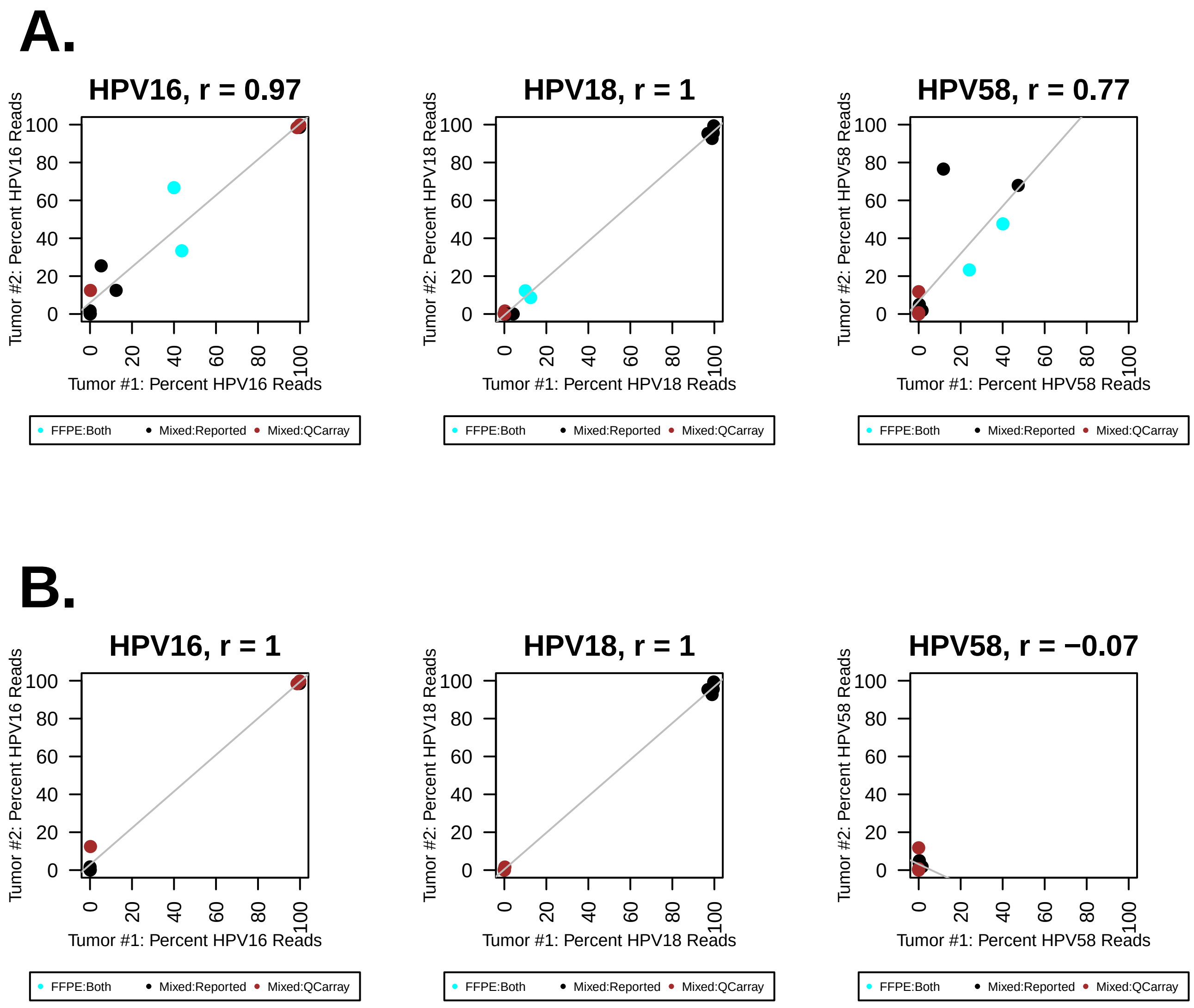
A.
HPV16, r = 0.97
HPV18, r = 1
HPV58, r = 0.77
B.
HPV16, r = 1
HPV18, r = 1
HPV58, r = −0.07
Tumor #2: Percent HPV16 Reads
Tumor #2: Percent HPV18 Reads
Tumor #2: Percent HPV58 Reads
Tumor #1: Percent HPV16 Reads
Tumor #1: Percent HPV18 Reads
Tumor #1: Percent HPV58 Reads
0
20
40
60
80
100
FFPE:Both
Mixed:Reported
Mixed:QCarray
