# Supplementary material for: Correction: HPV genotyping by L1 amplicon sequencing of archived invasive cervical cancer samples: a pilot study
Source: Infect Agent Cancer. 2024 Jun 19;19:28. doi: 10.1186/s13027-024-00589-0 (PMC11186234; doi:10.1186/s13027-024-00589-0)
Supplement: Supplementary file 1 — Additional file 1. [file 13027_2024_589_MOESM1_ESM.docx]

**Table S1: Reference Sequences Used for HPV Genotyping**

| **HPV Genotype** | **Accession**  **Number** | **Munoz et al. 2003 (Ref. 16)^a^** | **Van Doorslaer et al. 2010 (Ref. 17)^b^** | **Bouvard et al. 2009**  **(Ref. 14)^c^** |
| --- | --- | --- | --- | --- |
| HPV16 | K02718 | High Risk | Oncogenic Branch | Carcinogenic (Group 1) |
| HPV18 | NC_001357 | High Risk | Oncogenic Branch | Carcinogenic (Group 1) |
| HPV31 | J04353 | High Risk | Oncogenic Branch | Carcinogenic (Group 1) |
| HPV33 | M12732 | High Risk | Oncogenic Branch | Carcinogenic (Group 1) |
| HPV35 | M74117 | High Risk | Oncogenic Branch | Carcinogenic (Group 1) |
| HPV39 | KC470249 | High Risk | Oncogenic Branch | Carcinogenic (Group 1) |
| HPV45 | KC470260 | High Risk | Oncogenic Branch | Carcinogenic (Group 1) |
| HPV51 | KF436887 | High Risk | Oncogenic Branch | Carcinogenic (Group 1) |
| HPV52 | GQ472848 | High Risk | Oncogenic Branch | Carcinogenic (Group 1) |
| HPV56 | EF177181 | High Risk | Oncogenic Branch | Carcinogenic (Group 1) |
| HPV58 | D90400 | High Risk | Oncogenic Branch | Carcinogenic (Group 1) |
| HPV59 | X77858 | High Risk |  | Carcinogenic (Group 1) |
| HPV73 | KF436836 | High Risk | Oncogenic Branch | Possibly Carcinogenic (Group 2B) |
| HPV82 | AB027021 | High Risk | Oncogenic Branch | Possibly Carcinogenic (Group 2B) |
| HPV68 | KC470283 | High Risk | Oncogenic Branch | Possibly Carcinogenic (Group 2A) |
| HPV26 | NC_001583 | Probable High Risk | Oncogenic Branch | Possibly Carcinogenic (Group 2B) |
| HPV53 | NC_001593 | Probable High Risk | Oncogenic Branch | Possibly Carcinogenic (Group 2B) |
| HPV66 | HPU31794 | Probable High Risk | Oncogenic Branch | Possibly Carcinogenic (Group 2B) |
| HPV34 | NC_001587 |  | Oncogenic Branch | Possibly Carcinogenic (Group 2B) |
| HPV67 | HQ537781 |  |  | Possibly Carcinogenic (Group 2B) |
| HPV69 | KF436864 |  |  | Possibly Carcinogenic (Group 2B) |
| HPV97 | EF436229 |  |  | Possibly Carcinogenic (Group 2B) |
| HPV85 | NC_034616 |  |  | Possibly Carcinogenic (Group 2B) |
| HPV30 | KF436850 |  | Oncogenic Branch | Possibly Carcinogenic (Group 2B) |
| HPV6(b) | HQ537781 | Low Risk |  | Not Classifiable (Group 3) |
| HPV11 | FR872717 | Low Risk |  | Not Classifiable (Group 3) |
| HPV70 | U21941 | Low Risk | Oncogenic Branch | Possibly Carcinogenic (Group 2B) |
| HPV40 | KU298895 | Low Risk |  |  |
| HPV42 | GQ472847 | Low Risk |  |  |
| HPV43 | AJ620205 | Low Risk |  |  |
| HPV44 | U31788 | Low Risk |  |  |
| HPV54 | NC_001676 | Low Risk |  |  |
| HPV61 | NC_001694 | Low Risk |  |  |
| HPV72(b) | KJ145795 | Low Risk |  |  |
| HPV81 | AJ620209 | Low Risk |  |  |

^a^High risk” genotypes were those associated with an odds ratio for cervical cancer of at least 5.0 and a lower 95 percent confidence limit of at least 3.0, as well as those that were detected in more than 3 patients but in none of the controls. “Probable high risk” genotypes met the criteria for “high risk” but were detected in only 1–3 patients and none of the controls. “Low risk” genotypes were those with an odds ratio for cervical cancer of at least 1.0 and a lower 95 percent confidence limit of less than 3.0, as well as those that were detected in some of the controls but in none of the patients.

^b^"Oncogenic Branch" from the branch/clade of the E6 phylogenetic tree that contains some HPV types labeled as “Oncogenic” (see Figure 7 of Ref. 17, which incorrectly cites “Bouvard et al. 2010", referring to Bouvard et al. 2009 [Ref. 14]).

^c^The International Agency for Research on Cancer (IARC) Monograph Working Group reassessed the carcinogenicity of HPV genotypes and designated them “carcinogenic to humans” (Group 1), “probably carcinogenic to humans” (Group 2), “possibly carcinogenic” (Group 2B), or “not classifiable as to its carcinogenicity to humans” (Group 3), based on epidemiological evidence and mechanistic studies (see Table 2 of Ref. 14).
